# Cytokine measurements add value to clinical variables in predicting outcomes for *Staphylococcus aureus* bacteremia

**DOI:** 10.1186/s12879-021-06010-0

**Published:** 2021-04-05

**Authors:** Karen Tan, Emi Minejima, Mimi Lou, Wendy J. Mack, Paul Nieberg, Annie Wong-Beringer

**Affiliations:** 1 Department of Clinical Pharmacy, University of Southern California (USC) School of Pharmacy, 1985 Zonal Avenue, Los Angeles, CA 90089 USA; 2 Department of Preventive Medicine, Keck School of Medicine, University of Southern California, Los Angeles, USA; 3 Department of Medicine – Infectious Diseases, Huntington Hospital, Pasadena, USA; 4 Department of Pharmacy, Huntington Hospital, Pasadena, USA

## Abstract

**Background:**

We demonstrated that an early dysregulated cytokine response [high interleukin-10 to tissue necrosis factor (IL-10/TNF) ratio] predicted poor outcomes in patients with *Staphylococcus aureus* bacteremia (SAB). However, high interpatient variability in cytokine levels were observed. We grouped cytokine measurements in quartiles and assessed their additive value to clinical variables for predicting bacterial persistence and 30-day mortality in patients with SAB.

**Methods:**

A multicenter observational study was conducted in hospitalized patients with SAB. Medical charts were reviewed for relevant information. Blood samples were obtained for cytokine measurements by ELISA: interferon-gamma (IFNγ), interleukin (IL-1β, IL-6, IL-8, IL-10, IL-17) and tissue necrosis factor (TNF). Cytokine measurements were grouped into quartiles. Significant predictors for bacterial persistence and 30-day mortality were determined by multivariable logistic regression analysis. Area under the ROC curve (AUC) analysis was performed and predictive performance was compared between models with and without cytokine quartiles.

**Results:**

Among 606 patients with SAB, a subset of patients (*n* = 239) had Day 1 cytokine measurements and clinical data collected; of those, 53 (22%) had persistent bacteremia. Accounting for septic shock, the addition of either IL-10 (AUC 0.708) or TNF (AUC 0.714) quartiles measured on Day 1 improved model performance for predicting bacterial persistence. All patients had Day 4 cytokine measurements; 52 patients (8.5%) died within 30-days of SAB onset. Inclusion of either IL-10 (AUC 0.873) or TNF (AUC 0.879) quartiles, but not both, measured on Day 4 to the significant clinical predictors (coronary artery disease, Pitt bacteremia score ≥ 4, and septic shock) improved model performance for mortality.

**Conclusions:**

IL-10 or TNF levels falling within the range in the upper quartiles, when combined with clinical variables, improved model performance for predicting outcomes, and may potentially be used to support aggressive management and biomarker-guided studies to evaluate the benefit of adjunctive immunotherapy for SAB in the future.

**Supplementary Information:**

The online version contains supplementary material available at 10.1186/s12879-021-06010-0.

## Introduction

*Staphylococcus aureus* is a leading cause of bacteremia with mortality rates of up to 30% [1]. Risk factors for mortality in patients with *S. aureus* bacteremia (SAB) include older age, endovascular source of infection, and presence of sepsis [1]. In addition, in a large cohort study of nearly 900 patients with SAB, our group found that persistent bacteremia defined by positive blood cultures for 3 days or longer significantly predicted mortality and that each day of continued bacteremia increased risk of mortality by 16% [2]. Despite receipt of antimicrobial therapy with apparent in vitro susceptibility for at least 72 h, one third of the patients with SAB continued to have growth of *S. aureus* in the blood [3].

We, and others, have shown that serum levels of immune biomarkers, including interferon-gamma (IFNγ), interleukin (IL-1β, IL-6, IL-8, IL-10, IL-17) and tissue necrosis factor (TNF), differentiate between patients with SAB who developed persistent bacteremia or died compared to those with prompt bacterial clearance and survived [4–9]. Importantly, we have shown that a dysregulated host immune response, characterized by the predominance of anti-inflammatory over pro-inflammatory cytokine release during the early course of SAB significantly predicts persistence and 30-day mortality [5, 8]. Thus, these immune biomarkers, in conjunction with clinical variables, could serve as a prognostic tool for clinicians to identify SAB patients who are more likely to have worse outcomes and to guide management, that may include empiric combination antibiotic therapy or adjunctive immunotherapy.

The threshold concentration for a specific cytokine that correlates with poor outcome of SAB is currently unknown. Further complicating the interpretation of cytokine measurements, our group has observed high interpatient variability in cytokine concentrations during SAB. For a given cytokine, measurements could vary > 1000 fold between patients. Given the heterogeneity between individuals, we calculated quartile values for a panel of cytokines measured on Day 1 and Day 4 of SAB and tested the predictability of clinical factors, with or without inclusion of each cytokine value in quartile, for bacterial persistence and 30-day mortality, respectively. *We hypothesize that there is a set range of cytokine measurement that represents host immune response unique to a physiological insult (*e.g. *S. aureus* *bacteremia) and that the magnitude of response within that range represents a distinct immunophenotype predictive of outcome*. Our aims in this study are to: 1) describe the predictive value of cytokine quartiles for duration of bacteremia and mortality in patients with SAB in conjunction with clinical variables and 2) define a concentration range for cytokine levels that could guide clinical decisions.

## Methods

### Study design/setting/patients

We conducted a prospective, observational study from July 2012 to July 2018 at three affiliated medical centers in Los Angeles County, CA. Hospitalized patients were included if they met the following criteria: age ≥ 18 years, had monomicrobial *S. aureus* growth from at least one blood culture, received at least 48 h of effective antibiotic therapy, and had blood cytokine measurements on either Day 1 and/or Day 4 of SAB. Day 1 was defined as the day of the first positive blood culture with *S. aureus*, and Day 4 was 72 h thereafter. Patients were excluded if blood cultures were polymicrobial, serum samples not available for cytokine analysis, medical records were incomplete or unavailable, or anti-staphylococcal therapy was either delayed (> 48 h) or less than 48 h of therapy was administered.

### Clinical endpoints

Primary clinical endpoints were bacterial persistence and 30-day all-cause mortality. Bacterial persistence was defined as four or more consecutive days of positive blood cultures after initiation of anti-staphylococcal therapy [5]. Mortality was assessed for up to 30-days after the first positive *S. aureus* blood culture. Demographic data, including age, sex, and comorbid conditions, as well as antibiotic treatment details and receipt of chemotherapy or other immunosuppressive drugs were recorded. Microbiologic and laboratory results included culture and sensitivities, vital signs, complete blood cell count, comprehensive metabolic panel, and presence of sepsis or septic shock. Complicated bacteremia as defined by any one the following variables were documented: endocarditis, implanted prostheses, temperature of > 38.3 °C after 72 h of initiating effective therapy, and evidence of metastatic sites of infection. Time to procedure performed for source control was recorded. Pitt bacteremia score was calculated using the worst score within 48 h before or on the day of the first positive blood culture. qSOFA score was calculated based on three clinical variables worth 1 point each: systolic blood pressure ≤ 100 mmHg, respiratory rate ≥ 22 breaths/min, and Glasgow coma score < 15 or altered mental status noted by the treating physician [10]. Source of bacteremia was divided relative to risk of mortality as previously defined [11]: high (> 20%), intermediate (10–20%), and low (< 10%). Study data were collected and managed using REDCap electronic data capture tools hosted at University of Southern California [12, 13].

### Cytokine measurements

Convenience blood samples were obtained on Day 1 and/or Day 4 of SAB where available for cytokine analysis. Samples were centrifuged and stored at − 80 °C until further analyses. Cytokine concentrations (IFNγ, IL-1β, IL-6, IL-8, IL-10, IL-17, TNF) were measured in duplicates using Luminex multiplex assay (Millipore, Billerica, MA) per manufacturer’s instructions.

### Data analyses

To explore the predictive value of cytokine levels, cytokines were categorized by quartile values. Descriptive analyses were performed by either Student t test or Mann-Whitney U test for continuous variables and Fisher’s exact or chi-square test for categorical variables. Based on their measured cytokine response, patients were grouped into their respective quartile group for each cytokine and Bonferroni correction was applied for Chi-square test on the comparisons between any two quartiles among the study groups.

Univariate logistic regression was performed to assess the association of clinical variables and cytokine quartiles with bacterial persistence and 30-day mortality. To develop the predictive models for persistence and mortality, variables from the univariate analysis with a *p*-value < 0.20 were included in subsequent multivariable model selection. Calculated cytokine quartiles were then added individually into the multivariable model with clinical variables only. For the bacterial persistence model, Day 1 cytokine quartiles were considered as potential early biomarkers predictive of prolonged duration of bacteremia. Day 4 cytokine quartiles were explored in the 30-day mortality model, where therapeutic management could be re-evaluated in patients failing to respond after 72 h of antimicrobial therapy. Subgroup analysis on patients with MRSA and MSSA bacteremia was performed and multivariable models were developed separately. Receiver operating characteristics (ROC) and area under the ROC curve (AUC) analyses were generated to compare the predictive performance of the clinical base models versus cytokine-added models. Statistical analyses were performed using GraphPad Prism version 8.4.2 (San Diego, CA, USA) and SAS software, version 9.4 (SAS Institute Inc., Cary, NC, USA.) was used for univariate and multivariate logistic regression modeling.

## Results

### Overall population and cytokine quartiles

A total of 606 patients met study inclusion criteria and had either Day 1 or Day 4 serum samples saved (Table 1**)**. Study patients were mostly male (68%, 414/606), with a mean(±SD) age of 59 years (±16.2 years). MSSA was the cause of bacteremia in two thirds of the study patients (66%, 399/606). The timing of antibiotic initiation and the agents prescribed were similar between study groups. A majority of the patients (77%, 430/606) were already receiving or initiated on an antibiotic regimen with at least one agent having anti-staphylococcal activity on the day of first positive blood culture for *S. aureus;* in total, 97% of patients were initiated on anti-staphylococcal therapy by day 2 of bacteremia. The initial anti-staphylococcal agent most commonly prescribed was vancomycin (76%, 463/606), followed by a beta-lactam (11%), linezolid (5.1%), and daptomycin (3.1%). Nearly half of the patients (48%, 292/606) received an initial combination of vancomycin and a beta-lactam agent; a slightly higher proportion of patients with resolving compared to persistent bacteremia received the combination (50% vs 42%, *p* = 0.07). Nearly a quarter of the patients (24%, 144/606) developed persistent bacteremia with a median duration of 5 days (IQR: 4, 7) compared to 1 day (IQR: 1, 3) for those in the resolving group (*p* < 0.0001). Overall in-hospital 30-day mortality occurred in 9% of patients (52/606); however, mortality rate was 2-fold greater among those who developed persistence compared to those with prompt resolution (17% vs 5.8%, *p <* 0.0001; OR: 3.39, CI: 1.88, 6.02). Quartile values were calculated for each measured cytokine (pg/ml). The range of values for all cytokines was higher on Day 1 compared to Day 4 overall. (Table 2).

**Table 1:** Patient characteristics and outcome grouped by persistence and mortality

	Overall ***N*** = 606	Persistence (Day 4)			Mortality		
		Resolve (***n*** = 462)	Persist (***n*** = 144)	***p***-value	Survivor (***n*** = 554)	Non-survivor (***n =*** 52)	***p***-value
**Age (mean, std)**	59+/−16.2	58 +/− 16.5	60+/− 14.8	0.21	64+/− 15.2	58 +/− 16.2	0.03
**Male**	414 (68%)	321 (70%)	93 (65%)	0.31	383 (69%)	31 (60%)	0.11
**Black race**	64 (11%)	40 (8.7%)	24 (17%)	0.01	58 (11%)	6 (12%)	0.81
**Comorbidities**							
**Hypertension**	319 (53%)	231 (50%)	88 (61%)	0.02	289 (52%)	30 (58%)	0.47
**Hyperlipidemia**	133 (22%)	97 (21%)	36 (25%)	0.36	120 (22%)	13 (25%)	0.60
**Diabetes**	247 (41%)	177 (38%)	70 (49%)	0.03	225 (41%)	22 (42%)	0.88
**CHF**^**a**^	83 (14%)	66 (14%)	17 (12%)	0.49	67 (12%)	16 (31%)	0.001
**CAD**^**b**^	92 (15%)	66 (14%)	26 (18%)	0.29	75 (14%)	17 (33%)	0.001
**Immunosuppressed**^**c**^	43 (7.1%)	29 (6.3%)	14 (9.7%)	0.16	36 (6.5%)	7 (13%)	0.062
**Dialysis**^f^	125 (21%)	93 (20%)	32 (22%)	0.74	111 (20%)	14 (27%)	0.48
**Infection characteristics**							
**MRSA**^**d**^	207 (34%)	149 (32%)	58 (40%)	0.09	185 (33%)	22 (42%)	0.22
**Source of bacteremia**^**e,f**^				0.65			0.003
**High**	134 (22%)	99 (21%)	35 (24%)	0.49	115 (21%)	19 (37%)	0.01
**Intermediate**	349 (58%)	271 (59%)	78 (55%)	0.38	329 (59%)	20 (39%)	0.01
**Low**	121 (20%)	91 (20%)	30 (21%)	0.72	109 (20%)	12 (24%)	0.58
**Procedure performed for source control**^f^	280 (46%)	212 (46%)	68 (48%)	0.77	264 (48%)	16 (31%)	0.02
**Complicated SAB**							
Endocarditis	48 (7.9%)	25 (5.4%)	23 (16%)	< 0.001	42 (7.6%)	6 (12%)	0.29
Implanted prostheses	221 (37%)	166 (36%)	55 (38%)	0.62	201 (36%)	20 (39%)	0.77
Tmax > 38.3 at 72 h after abx^f^	48 (10%)	26 (7.4%)	22 (18%)	0.0015	38 (8.8%)	10 (27%)	0.002
Metastatic complication^f^	118 (20%)	77 (17%)	41 (28%)	0.0026	104 (19%)	14 (27%)	0.20
**Initial regimen vancomycin with beta-lactam**	292 (48%)	232 (50%)	60 (42%)	0.07	266 (48%)	26 (50%)	0.07
**Pitt Bacteremia Score (median, IQR)**^f ^	1 (0,2)	1 (0, 2)	1 (0, 3)	0.19	1 (0, 2)	4 (2, 6)	< 0.0001
**< 4**	517 (87%)	395 (87%)	122 (87%)	Ref	493 (90%)	24 (47%)	Ref
≥ **4**	79 (13%)	60 (13%)	19 (13%)	> 0.99	52 (10%)	27 (53%)	< 0.0001
**qSOFA score (median, IQR)**^f^	1 (0,2)	1 (0,2)	1 (1,2)	0.21	1 (0, 2)	2 (1, 2)	< 0.0001
**< 2**	365 (61%)	286 (62%)	79 (56%)	Ref	351 (64%)	14 (27%)	Ref
≥ **2**	236 (39%)	173 (38%)	63 (44%)	0.17	198 (36%)	38 (73%)	< 0.0001
**Sepsis**^f^	490 (81%)	365 (80%)	125 (87%)	0.051	441 (80%)	49 (94%)	0.009
**Severe sepsis**^f^	257 (42%)	179 (40%)	78 (55%)	0.002	214 (39%)	43 (83%)	< 0.0001
**Septic shock** ^f^	70 (12%)	43 (9.5%)	27 (19%)	0.004	46 (8.4%)	24 (46%)	< 0.0001
**Required ICU stay**^f^	240 (40%)	169 (37%)	71 (50%)	0.008	192 (35%)	48 (92%)	< 0.0001
**Duration of SAB (median, IQR)**	1 (1,3)	1 (1,2)	5 (4, 7)	< 0.0001	1 (1,3)	3 (1, 5)	0.004
**30-day mortality**	52 (8.6%)	27 (5.8%)	25 (17%)	< 0.0001	0	52 (100%)	–

^**a**^**CHF:** congestive heart failure, ^b^**CAD**: coronary artery disease, ^**c**^**Immunosuppressed**: receipt of cancer chemotherapy or other immunosuppressive therapies, ^d^**MRSA**: methicillin-resistant *S. aureus*. ^e^Source definitions for mortality risk: Low-risk sources - intravenous catheter, urinary tract infection, ear-nose-larynx, gynecologic sources, and several manipulation-related sources (including digestive endoscopy, arterial catheterization, and sclerosis of esophageal varices); intermediate-risk sources - osteoarticular, soft-tissue, and unknown sources; high-risk sources - endovascular sources, lower respiratory tract, intraarticular, and central nervous system foci

*Ref* Reference

^f^Number of patients included for: **Dialysis (**Resolve 461, Persist 144; Survivor 553, Non-survivor 52), **Source of bacteremia** (Resolve 461, Persist 143; Survivor 553, Non-survivor 51), **Source control** (Resolve 460, Persist 143; Survivor 551, Non-survivor 52), **Tmax > 38.3 at 72 h after antibiotics** (Resolve 350, Persist 121; Survivor 434, Non-survivor 37), **Metastatic complication** (Resolve 461, Persist 144; Survivor 553, Non-survivor 52), **Pitt Bacteremia Score (**Resolve 455, Persist 141; Survivor 545, Non-survivor 51), **qSOFA score (**Resolve 459, Persist 142; Survivor 549, Non-survivor 52), **Sepsis (**Resolve 459, Persist 144; Survivor 551, Non-survivor 52), **Severe sepsis (**Resolve 452, Persist 143; Survivor 543, Non-survivor 52), **Septic shock (**Resolve 455, Persist 144; Survivor 547, Non-survivor 52), **Required ICU stay (**Resolve 458, Persist 143; Survivor 549, Non-survivor 52),

**Table 2:** Quartile values of cytokine measurements on Day 1 and Day 4 of SAB

Cytokine (pg/ml)	Quartile 1	Quartile 2	Quartile 3	Quartile 4	Overall ***p***-value Persistence	Overall ***p***-value Mortality
**TNF**						
Day 1	< 9.54	9.54–18.96	18.97–36.85	> 36.85	< 0.0001	
Day 4	< 6.73	6.73–12.57	12.58–24.67	> 24.67		< 0.0001
**IL-10**						
Day 1	< 11.11	11.11–29.96	29.97–118.22	> 118.22	0.002	
Day 4	< 5.49	5.49–13.57	13.58–28.93	> 28.93		< 0.0001
**IL-6**						
Day 1	< 25.92	25.92–69.56	69.57–222.65	> 222.65	0.10	
Day 4	< 7.02	7.02–19.93	19.94–57.20	> 57.20		0.001
**IL-8**						
Day 1	< 18.00	18.00–48.10	48.11–128.56	> 128.56	0.06	
Day 4	< 12.94	12.94–28.05	28.06–68.95	> 68.95		< 0.0001
**IFNγ**						
Day 1	< 4.34	4.34–9.13	9.14–18.69	> 18.69	0.19	
Day 4	< 3.45	3.45–7.00	7.01–13.25	> 13.25		0.69
**IL-1β**						
Day 1	< 1.21	1.21–2.77	2.78–3.25	> 3.25	0.50	
Day 4	< 0.83	0.83–1.84	1.85–3.64	> 3.64		0.87
**IL-17**						
Day 1	< 1.92	1.92–5.07	5.08–11.22	> 11.22	0.17	
Day 4	< 1.88	1.88–3.75	3.76–9.88	> 9.88		0.23

All cytokine concentrations (IFNγ, IL-1β, IL-6, IL-8, IL-10, IL-17, TNF) were measured in duplicates using Luminex multiplex assay (Millipore, Billerica, MA) per manufacturer’s instructions. Chi-square (or Fisher’s exact) tests were performed on Day 1 cytokine measurements for persistence and Day 4 measurements for mortality, respectively

### Predictors of bacterial persistence

#### Clinical variables

Patients with bacterial persistence were more likely to be black (17% vs 8.7%, *p* = 0.01), have a diagnosis of hypertension (61% vs 50%, *p* = 0.02) and diabetes (49% vs 38%, *p* = 0.03) (Table 1). A higher proportion of patients with persistent SAB had a diagnosis of endocarditis (16% vs 5.4%, *p* < 0.001), remained febrile (> 38.3 °C) after 72 h of antibiotic therapy (18% vs 7.4%, *p* = 0.0015), and met criteria for either severe sepsis (55% vs 40%, *p* = 0.002) or septic shock (19% vs 9.5%, *p* = 0.004) at the time of SAB onset; nearly half required an ICU stay during the course of SAB compared to 37% of those with prompt bacterial clearance (*p* = 0.008). Metastatic complications were more common in persistent infections (28% vs 17%; *p* = 0.0026). A trend towards more patients in the persistent group had MRSA as the causative pathogen of bacteremia was observed (40% vs 32%, *p* = 0.09). Patients with persistent bacteremia were less likely to have received an initial combination regimen of vancomycin with a beta-lactam (42%, vs 50%, *p* = 0.07).

#### Day 1 cytokines

**Fig. 1:**
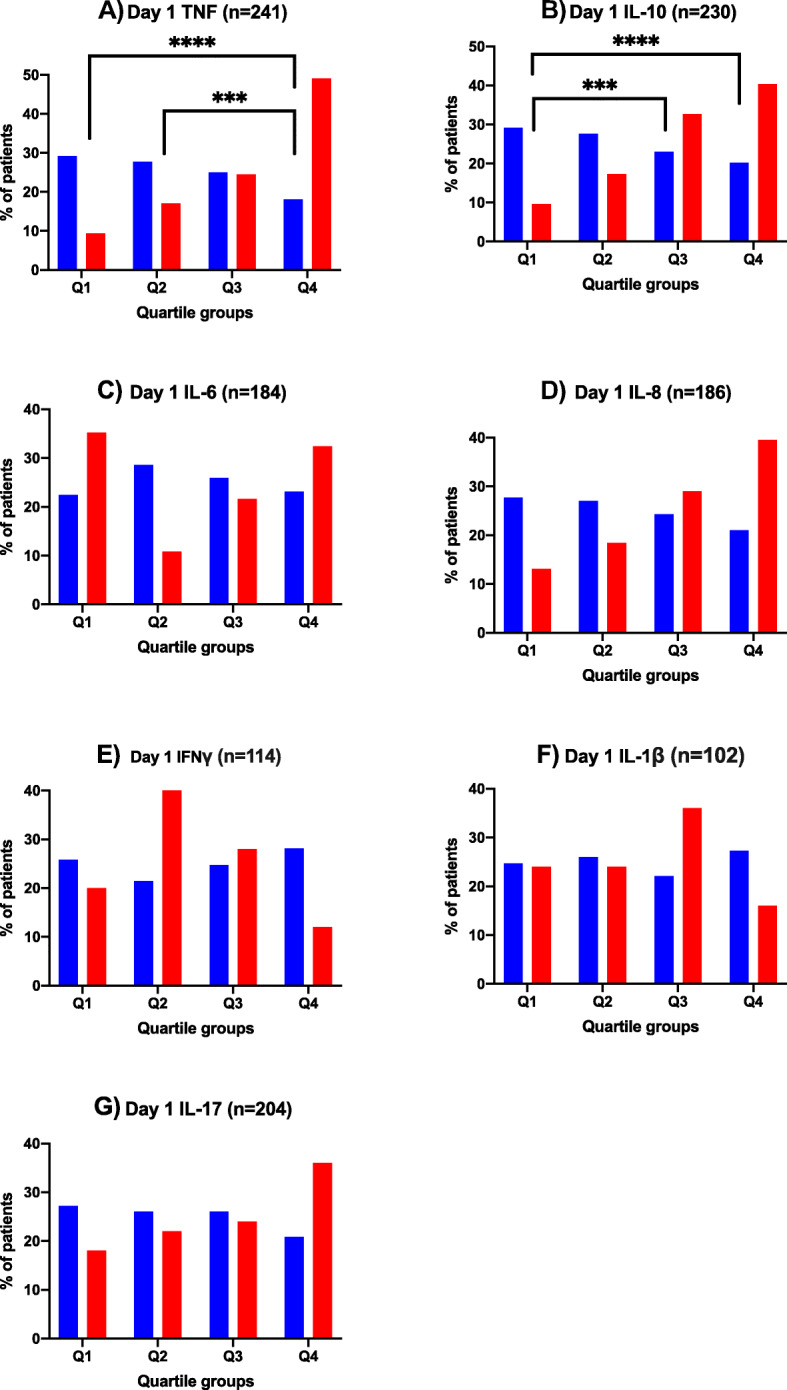
Distribution of Patients across Cytokine Quartiles at Baseline (Day 1) Comparing Resolving vs Persistent SAB Groups. Overall differences between groups (persistent, red vs resolving, blue) was calculated by chi-square analyses for TNF (*n =* 53 vs *n* = 188), IL-10 (*n =* 52 vs *n* = 178), IL-6 (*n* = 37 vs *n* = 147), IL-8 (*n =* 38 vs *n* = 148), IFNγ (*n =* 25 vs *n* = 89), IL-1β (*n* = 25 vs *n* = 77), IL-17 (*n =* 50 vs *n* = 154). Then, Bonferroni correction was made for multiple pairwise comparisons, with a threshold for significance of *p* < 0.008 (0.05/6). Significant *p*-values are listed in the figure. Note, *P* ≤ 0.001 is denoted by ***, and *P* ≤ 0.0001 is denoted by ****

Cytokine measurements on Day 1 were available for a subset of study patients. The number of patients for whom Day 1 levels for respective cytokines were available are listed in descending order as follows: TNF (*n* = 241; 53 persistent), IL-10 (*n* = 230; 52 persistent), IL-17 (*n* = 204; 50 persistent), IL-8 (*n* = 186; 38 persistent), and IL-6 (*n* = 184; 37 persistent), IFNγ (*n* = 114; 25 persistent), IL-1β (*n* = 102; 25 persistent). In this subset of patients with a measured cytokine response at onset of bacteremia, the proportion of patients with persistent vs. resolving bacteremia significantly differed by Day 1 quartiles of TNF (*p* < 0.0001) and IL-10 (*p* = 0.002) measurements; a trend towards significance was observed for Day 1 quartiles of IL-6 (*p* = 0.10) and IL-8 (*p* = 0.06). No significant association was identified between persistent and resolving bacteremia by Day 1 IFNγ (*p* = 0.19), IL-1β (0.50), and IL-17 (*p* = 0.17) quartiles (Table 2) and were therefore not examined further in the multivariate model for persistence. Comparisons between any two quartiles revealed significant differences for patients with resolving vs persistent SAB with higher proportion of the latter group showing TNF response in Quartile 4 than Quartile 1 or 2 whereas more persistent patients showed a Quartile 3 or 4 response than Quartile 1 for IL-10 (Fig. 1).

#### Regression model

In the subset of 239 patients who had at least one cytokine measured at onset of bacteremia (Day 1), we first performed a univariate analysis to identify which clinical variables (Table 1) and Day 1 cytokine quartile groups (Table 2) significantly predicted bacterial persistence. By univariate logistic regression, the following variables were identified as significant: black race, hypertension, Tmax > 38.3 °C after 72 h of antimicrobial therapy, documented metastatic complication, initial regimen with vancomycin with a beta-lactam, sepsis, severe sepsis, septic shock, Pitt bacteremia score, and qSOFA score ≥ 2. Sepsis, severe sepsis, and septic shock were colinear; therefore, we only considered septic shock for inclusion as it was the most significant predictor of the three variables. Similarly, septic shock and ICU stay were colinear with septic shock remaining the most significant predictor. A strong correlation between Tmax after 72 h of antimicrobial therapy and persistence was observed; however, because this model focused on utilizing variables collected at onset of SAB infection, these variables were not included for further testing.

Using multivariable logistic regression, septic shock was identified in the clinical base model as the single independent predictor for bacterial persistence (OR 4.09, 95% CI 1.826, 9.173) with a predictive performance at AUC 0.601 (95% CI 0.537, 0.664) (Table 3).

**Table 3:** Multivariable predictive model for persistence: Clinical variables alone and added predictive value of day 1 cytokines

Model (Sample Size)	Odds Ratio (95% CI)	***p***-value	AUC (95% CI)	***p-***value for AUC difference from clinical model
**Clinical variable model (239)**				
Septic shock at onset of SAB	4.09 (1.826, 9.173)	< 0.001	0.601 (0.537, 0.664)	–
**Added Day 1 cytokine variables individually as quartiles** ^**a**^**:**				
**TNFα (239)**			**0.714 (0.635, 0.794)**	**0.0005**
Q1: < 9.54	Ref			
Q2: 9.54–18.96	1.92 (0.6, 6.142)	0.27		
Q3: 18.97–36.85	2.79 (0.918, 8.475)	0.07		
Q4: > 36.85	6.79 (2.334, 19.758)	0.0004		
**IL-10 (228)**			**0.708 (0.629, 0.788)**	**0.001**
Q1: < 11.11	Ref			
Q2: 11.11–29.96	1.92 (0.599, 6.139)	0.27		
Q3: 29.97–118.22	3.80 (1.274, 11.271)	0.02		
Q4: > 118.22	4.53 (1.487, 13.808)	< 0.01		
**IL-8 (184)**			**0.666 (0.568, 0.765)**	**0.02**
Q1: < 18.00	Ref			
Q2: 18.00–48.10	1.27 (0.369, 4.372)	0.70		
Q3: 48.11–128.56	2.09 (0.642, 6.802)	0.22		
Q4: > 128.56	3.60 (1.163, 11.123)	0.03		
**IL-6 (182)**			**0.600 (0.486–0.715)**	**0.53**
Q1: < 25.92	Ref			
Q2: 25.92–69.56	0.22 (0.064, 0.745)	0.02		
Q3: 69.57–222.65	0.44 (0.157, 1.235)	0.12		
Q4: > 222.65	0.72 (0.27, 1.912)	0.51		

^a^Only included cytokines contributing significant prediction beyond clinical variable model; *Q* quartile, *Ref* reference

Significant improvements in model performance occurred with the addition of either Day 1 TNF (*p* = 0.0005) or Day 1 IL-10 (*p* = 0.001) quartiles. In the model with Day 1 TNF quartiles added (AUC 0.714, 95% CI 0.635, 0.794), patients with a TNF response in quartile 4 (> 36.85 pg/ml) had 5.8 times greater risk for developing persistence (OR 6.79, 95% CI 2.334, 19.758, *p* < 0.01). Likewise, the inclusion of Day 1 IL-10 quartiles improved model AUC to 0.708 (95% CI 0.629,0.788). Patients with a Day 1 Quartile 3 (29.97–118.22 pg/ml) IL-10 response were 2.8 times more likely to develop persistence compared to those with a Quartile 1 response (OR 3.80, 95% CI 1.274, 11.271, *p* = 0.02). The risk increased further to 3.5 times for those with a IL-10 Quartile 4 (> 118.22 pg/ml) response (OR 4.53, 95% CI 1.487,13.808, *p <* 0.01). The addition of Day 1 IL-8 quartiles slightly improved model performance (AUC 0.666, 95% CI 0.568, 0.765), however septic shock was no longer significant (OR 2.514, 95% CI 0.909–6.955, *p* = 0.07). No improvement was seen when Day 1 IL-6 quartile measures were included in the clinical model (AUC 0.600, 95% CI 0.486, 0.715).

### Predictors of mortality

#### Clinical variables

Overall mortality at 30-day following onset of SAB occurred in 9% of patients (52/606). Demographics and clinical presentation are detailed in Table 1. Interestingly, predisposing factors for persistence were not significantly associated with risk for death. Patients who died were more likely to have underlying cardiovascular disease (congestive heart failure 31% vs 12%, *p* = 0.001; coronary artery disease: 33% vs 14%, *p =* 0.001) and more severe disease at presentation such as higher median Pitt Bacteremia Score (4 vs 1, *p* < 0.0001) and greater proportion had qSOFA score of 2 or higher (73% vs 36%, *p <* 0.0001), severe sepsis (83% vs 39%, *p <* 0.0001) or septic shock (46% vs 8.4%, *p <* 0.0001), required admission to intensive care unit (92% vs 35%, *p <* 0.0001), and remained febrile after 72 h of antibiotic therapy (27% vs 8.8%, *p <* 0.002). Non-survivors were less likely to have had documented source control (31% vs 48%, *p* = 0.02) compared to survivors.

#### Day 4 cytokine

**Fig. 2:**
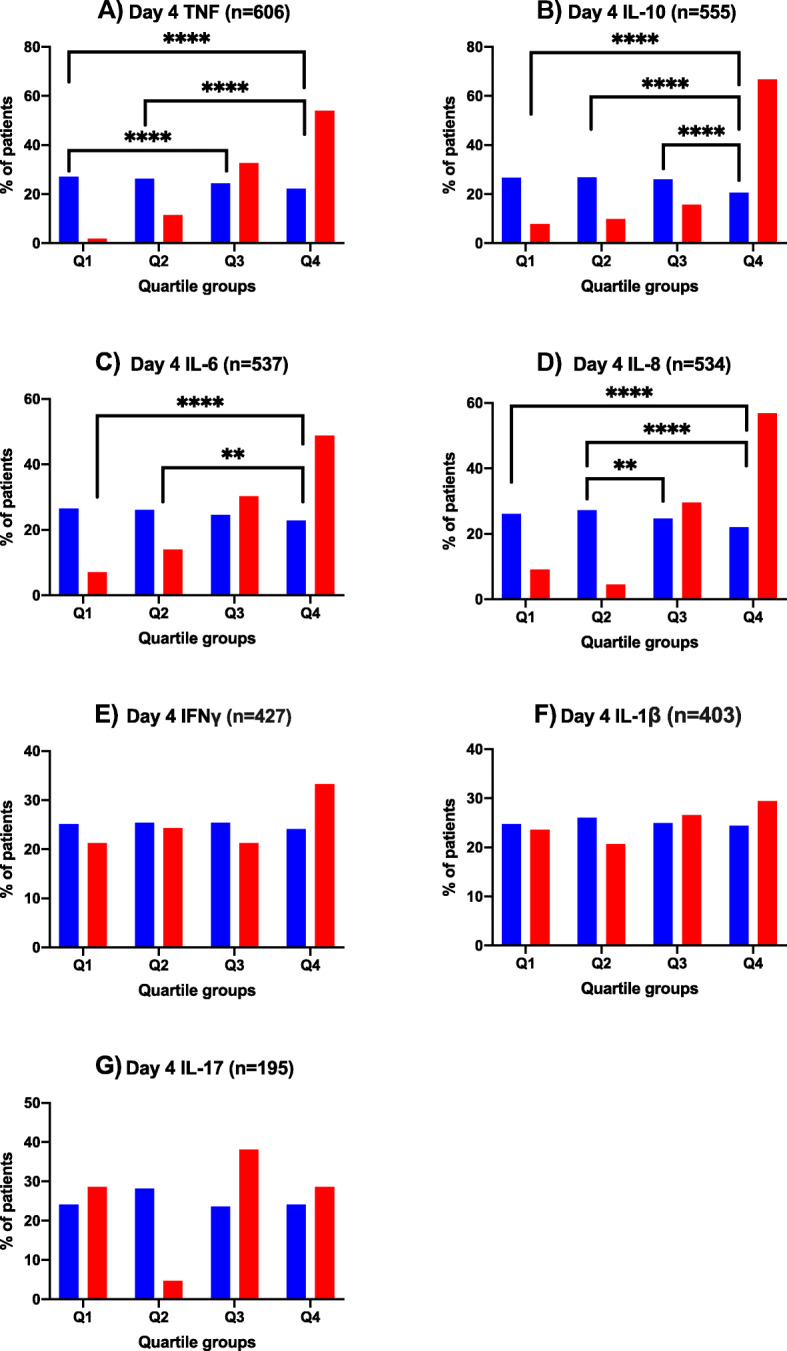
Distribution of Patients across Cytokine Quartiles on Day 4 of bacteremia Comparing Survivors vs Non-survivors. Overall differences between groups (non-survivor, red vs survivor, blue) was calculated by chi-square analyses for TNF (*n* = 52 vs *n* = 554), IL-10 (*n* = 51 vs *n* = 504), IL-6 (*n* = 43 vs *n* = 494), IL-8 (*n* = 44 vs *n* = 490), IFNγ (*n* = 33 vs *n* = 394), IL-1β (*n* = 34 vs *n* = 369), IL-17 (*n* = 21 vs *n* = 174). Then, Bonferroni correction was made for multiple pairwise comparisons, with a threshold for significance of *p* < 0.008 (0.05/6). Significant *p*-values are listed in the figure. Note, *P* ≤ 0.01 is denoted by **, *P* ≤ 0.001 is denoted by ***, and *P ≤* 0.0001 is denoted by ****

All 606 patients (554 survivor, 52 non-survivor) had Day 4 TNF measurements. Other Day 4 measurements were available for the following cytokines: IL-6 (*n* = 537 patients; 43 non-survivors), IL-8 (534 patients; 44 non-survivors), IL-10 (555 patients; 51 non-survivors), IFNγ (427 patients; 33 non-survivors), IL-1β (403 patients; 34 non-survivors), and IL-17 (195 patients; 21 non-survivors). Similar to that observed in patients with persistent bacteremia, non-survivors with measured cytokine response on Day 4 of bacteremia significantly differed from survivors in overall TNF, IL-10 and IL-8 response (Table 2). Additionally, overall IL-6 response also differed between study groups for survival analysis. Comparison of Day 4 cytokine quartile groups showed a significantly increasing proportion of non-survivors with responses in the higher quartiles especially Quartile 4 for TNF, IL-10, IL-6, and IL-8. (Fig. 2) Cytokine response on Day 4 for IFNγ, IL-1β, and IL-17 did not differ between those who died or survived and were therefore not tested further by adding to the multivariable model with clinical variables.

#### Regression model

The following variables were tested for model inclusion: age, sex, congestive heart failure, coronary artery disease, source of bacteremia, documented source control, Tmax > 38.3 °C after 72 h of antimicrobial therapy, septic shock, Pitt bacteremia score ≥ 4, qSOFA score ≥ 2. Sepsis, severe sepsis, and requirement of an ICU stay were also evaluated but not included to account for collinearity. By multivariable logistic regression analysis, the following clinical variables were identified as independent predictors for 30-day mortality: coronary artery disease (OR 2.93, 95% CI 1.42, 6.08), septic shock (OR 4.92, 95% CI 2.35, 10.29), and a Pitt bacteremia score ≥ 4 (OR 5.44, 95% CI 2.67, 11.09). Receiver operating curves for the clinical model showed an AUC of 0.804 (95% CI: 0.735, 0.873). By univariate analyses, Day 4 cytokine quartiles for IL-6, IL-8, TNF, and IL-10 were identified as significant predictors of mortality and were therefore added individually to the clinical model (Table 4).

**Table 4:** Multivariable predictive model for 30-day mortality: Clinical variables, and added predictive value of day 4 cytokines

Model (Sample Size)	Odds Ratio (95% CI)	***p***-value	AUC (95% CI)	***p-***value for AUC difference from clinical model
**Clinical variable model (576)**				
CAD	2.93 (1.42, 6.08)	0.004		
Septic shock at onset of SAB	4.92 (2.35, 10.29)	< 0.0001		
PBS ≥4 (vs. < 4)	5.44 (2.67, 11.09)	< 0.0001		
			0.804 (0.735, 0.873)	–
**Added Day 4 cytokine variables individually as quartiles** ^**a**^**:**				
**TNFα (581)**			**0.879 (0.838, 0.920)**	**0.0003**
Q1: < 6.73	Ref			
Q2: 6.73–12.57	6.89 (0.77, 61.75)	0.73		
Q3: 12.58–24.67	21.63 (2.69, 173.82)	< 0.01		
Q4: > 24.67	27.7 (3.52, 217.73)	0.0003		
**IL-10 (531)**			**0.873 (0.817, 0.930)**	**0.003**
Q1: < 5.49	Ref			
Q2: 5.49–13.57	1.71 (0.41, 7.24)	0.32		
Q3: 13.58–28.93	2.38 (0.63, 8.92)	0.82		
Q4: > 28.93	10.74 (3.28, 35.14)	< 0.0001		
**IL-6 (513)**			**0.828 (0.765, 0.892)**	**0.03**
Q1: < 7.02	Ref			
Q2: 7.02–19.93	1.53 (0.34, 6.98)	0.21		
Q3: 19.94–57.20	4.12 (1.07, 15.91)	0.12		
Q4: > 57.20	6.45 (1.74, 23.97)	0.001		
**IL-8 (510)**			**0.846 (0.783, 0.908)**	**0.01**
Q1: < 12.94	Ref			
Q2: 12.94–28.05	0.59 (0.10, 3.54)	0.03		
Q3: 28.06–68.95	3.51 (0.10, 12.34)	0.11		
Q4: > 68.95	7.87 (2.40, 25.82)	< 0.0001		

^a^Only included cytokines contributing significant prediction beyond clinical variable model; *Q* quartile, *Ref* reference

Inclusion of the aforementioned Day 4 cytokines to the clinical model significantly changed model performance: IL-6 (*p* = 0.03), IL-8 (*p* = 0.01), TNF (*p* = 0.0003), and IL-10 (*p* = 0.003). The greatest improvements in AUC were observed with the addition of Day 4 TNF and Day 4 IL-10 quartiles. The addition of Day 4 TNF quartiles improved the AUC from 0.804 to 0.879 (95% CI 0.838, 0.920). Risk for mortality increased for patients who expressed either a Quartile 3 (12.58 to 24.67 pg/ml) or Quartile 4 (> 24.67 pg/ml) TNF response. Compared to a TNF quartile 1 (< 6.73 pg/ml) response, patients with a Quartile 3 and 4 response had a 20-fold (OR 21.63, 95% CI 2.69, 173.82) and a 27-fold higher risk for death (OR 27.7, 95% 3.52, 217.73), respectively. Improvements in AUC were also seen with the inclusion of Day 4 IL-10 quartiles to clinical variables (AUC 0.873, 95% CI 0.817, 0.930). After controlling for the significant clinical variables, patients who expressed a Day 4 IL-10 response in quartile 4 (> 28.93 pg/ml) had 9.7 times greater risk (OR 10.74, 95% CI 3.28, 35.14) for mortality compared to those with a quartile 1 (< 5.49 pg/ml) response. Model performance was also improved with the addition of Day 4 IL-6 and Day 4 IL-8 quartiles, where patients with a quartile 4 response (> 57.2 pg/ml, > 68.95 pg/ml, respectively) had about a 6 times greater risk for mortality compared to patients with a quartile 1 response (< 7.02 pg/ml, < 12.94 pg/ml, respectively).

### Subgroup analysis: MRSA vs MSSA

An exploratory subgroup analysis comparing patients with MRSA to MSSA bacteremia was performed. Patients with MRSA bacteremia were more likely to have had endocarditis (16% vs 5.4%, *p* < 0.001) and a Tmax > 38.3 °C after 72 h of antimicrobial therapy (18% vs 7.4%, *p* = 0.0015) compared to patients with MSSA bacteremia. Patients with an MRSA infection were less likely to have received combination therapy with vancomycin and beta-lactam (75% vs 87%, *p* = 0.0041). A higher proportion of patients with MRSA bacteremia presented with septic shock on Day 1 of SAB (15% vs 9.8%, *p* = 0.060) and developed a metastatic complication (28% vs 17%, *p* = 0.0026). However, no significant differences in the rate of persistent bacteremia (28% vs 22%, *p* = 0.066) and 30-day mortality (11% vs 7.5%, *p* = 0.220) were observed when comparing patients with MRSA versus those with MSSA bacteremia, respectively.

We examined the cytokine profile of IL-10 and TNF as those were identified to be the most predictive, along with clinical variables, for bacterial persistence (Day 1) and mortality (Day 4). For patients with an MSSA bacteremia who had Day 1 cytokine measurements (*n* = 159), a clinical base model including only septic shock had a poor model performance for predicting bacterial persistence (AUC = 0.579, 95% CI 0.506, 0.651). The addition of either Day 1 IL-10 (AUC 0.725, 95% CI 0.628, 0.821) or TNF (AUC 0.722, 95% CI 0.625, 0.818) quartiles significantly improved the model; however, septic shock was no longer a significant predictor of persistence. (Appendix Table 1) Separately for MRSA bacteremia, there were 81 patients who had Day 1 measurements of either IL-10 and/or TNF. Adding Day 1 IL-10 or TNF quartiles to the clinical model did not result in any significant changes. (Data not shown).

For the mortality models, a total of 309 patients with MSSA bacteremia had Day 4 cytokine measurements available. The clinical model that included septic shock, Pitt bacteremia score ≥ 4, and Tmax > 38.3 °C after 72 h of antimicrobial therapy retained good model performance (AUC = 0.798, 95% CI 0.696, 0.901). The addition of either Day 4 IL-10 (AUC = 0.878, 95% CI 0.819, 0.936) or Day 4 TNF (AUC = 0.880, 95% CI 0.825, 0.935) quartiles significantly improved model performance, where patients with a quartile 4 cytokine response (IL-10: > 28.21 pg/ml, TNF: > 23.75 pg/ml) were more likely to die compared to patients with a quartile 1 response. (Appendix Table 2) In comparison, a total of 207 patients with MRSA infection had Day 4 cytokine measurements available. Since no deaths occurred among those who had quartile 1 response for IL-10 and TNF, we modified our model development by using quartile 2 response as our reference group for mortality analysis. Using the clinical model which included Pitt bacteremia score ≥ 4 and septic shock (AUC = 0.801, 95% CI 0.690, 0.912), and quartile 2 as the reference group, the addition of Day 4 IL-10 quartiles (*p* = 0.03), but not Day 4 TNF quartiles (*p* = 0.07), significantly improved model performance. Patients with MRSA bacteremia, septic shock, Pitt bacteremia score of ≥4, and a quartile 4 IL-10 response (> 31.13 pg/ml) were 5 times more likely to die (OR 6.39, 95% CI 1.11, 36.74) compared to those with similar clinical characteristics who had a quartile 2 IL-10 response (5.15–13.57 pg/ml). (Appendix Table 3).

## Discussion

Elevated cytokine measurements (e.g. IL-10) during *S. aureus* bacteremia (SAB) have been shown to significantly predict poor clinical outcomes, including persistent infection [4–6], all-cause mortality [4–7, 9], and complicated SAB [14], but the concentration range that defines an “elevated” measurement remains unclear. Here, we grouped the cytokine levels measured from a large cohort of patients with confirmed SAB into quartiles, then determined the predictive value of cytokine quartile groups, when combined with clinical variables, for bacterial persistence and mortality as the outcome endpoints using univariate and multivariable regression analysis. Our study found that the addition of either IL-10 or TNF quartile groups to clinical variables improved model prediction for both bacterial persistence and 30-day mortality.

Studies describing the association between cytokines and persistence in patients with SAB have used varying definitions to define bacterial persistence, ranging from 3 to 7 consecutive days of positive blood cultures [4, 6]. Additionally, differences in blood sample collection times were noted across studies. We used the persistence definition of ≥4 days of consecutive *S. aureus* blood cultures and found the ratio of IL-10/TNF levels measured on day 1 of SAB to be the most predictive [5], whereas others who defined persistence as > 4 days reported IL-10 [4] and IL-17A [6] as a significant predictors. For the studies using a persistence definition of > 4 days, sample collection times differed between the studies and were collected at either Day 1 of SAB or within 2 days of empiric antibiotic initiation, respectively. Using a definition of ≥4 days, our current study identified IL-10 and TNF quartiles, but not IL-17A, measured on Day 1 to be the most predictive for persistent bacteremia. Interestingly, the source of SAB infection may also affect cytokine production. In a study that reported IL-17A as the most predictive marker for persistent bacteremia, baseline levels were also noted to be highest in patients with an endovascular source [6]. In contrast, among those with a baseline cytokine measurement in our study population, only 18% had an endovascular source of infection while most had skin and skin structure infections which may explain why IL-17 was not found to be a significantly predictor in our cohort of SAB patients. Differences in sample collection time may have also contributed to variations in significant cytokines observed since different cytokines have varying peak times during the course of infection.

With respect to 30-day mortality, Day 1 IL-10 levels [4] and Day 4 IL-10/TNF ratio [5] were previously reported to be significant predictors, whereas IL-8 and CCL2 levels measured on Day 1–3 of empiric antibiotic therapy were associated with 90-day all-cause mortality [6]. In this study, the strongest predictive model for 30-day mortality was one that included clinical variables with either IL-10 or TNF quartiles collected on Day 4 of SAB. Others have reported that complicated SAB is associated with significantly higher baseline IL-6 levels compared to uncomplicated bacteremia. Our study found the most predictive model for 30-day mortality is one that incorporates either IL-10 or TNF quartiles collected on Day 4 of SAB with clinical variables. It is also possible that IL-8 can be used to predict late deaths in patients with SAB, rather than deaths occurring within 30 days of bacteremia onset. Others also found that Day 1 IL-6 levels were significantly higher in patients with a complicated infection when compared to uncomplicated SAB [14]. Here, we observed an association between IL-6 quartiles and mortality. While significant, IL-6 did not add to the predictive value of the model based on clinical variables alone. Taken together, it appears that outcome definitions (i.e. duration of bacteremia used to define persistence), the source of bacteremia, and the timing of sample collection may impact which cytokines are predictive of outcome.

While the association between cytokines and bacterial persistence or mortality have been previously described, the actual cutoff value used to discriminate between patients with SAB at risk for poor outcomes is unclear. It is worth noting that a “normal” level defined by reference laboratories is typically based on data obtained from healthy volunteers. In a study that collected serial samples from patients meeting sepsis-3 criteria until discharge [15], differences in cytokine measurements on Day 1 of sepsis were seen, where IL-1β, IL-10, IL-6, and IL-8 levels were among six cytokines found to be significantly elevated compared to controls. Beyond Day 1 of sepsis, both IL-6 and IL-8 remained significantly higher than controls. Other cytokine biomarkers, including IFNγ, IL-17A, TNF, were also evaluated but no differences were noted between study groups. Thus, an elevated cytokine measurement above the normal range is consistent with an active infectious disease process but does not differentiate between those who are at risk for poor outcome in patients with sepsis due to all causes.

Bacterial infections caused by gram negative compared to gram positive bacteria appear to elicit different cytokine responses [16]. Accordingly, infection-specific cytokine breakpoints will be important for identifying patients at high risk for poor outcomes. In this study focusing on patients with *S. aureus* bacteremia, we found IL-10 and TNF quartile groups to be the most predictive for persistence and 30-day mortality in patients with SAB. High interpatient variability in cytokine response was observed among patients with levels ranging from 1.36 to 2335.45 pg/ml for TNF and 0.07 to 2836.60 pg/ml for IL-10 on Day 1 of SAB. We therefore grouped cytokine measurements on Day 1 and Day 4 into Quartiles for comparison between those with persistent bacteremia vs prompt clearance and between those who died vs survived. Specifically, cytokine levels that fell within the ranges for Quartile 1 and Quartile 4 respectively on Day 1 of SAB were: IL-10 (< 11.11 pg/ml; > 118.22 pg/ml), TNF (< 9.54 pg/ml; > 36.85 pg/ml). The findings from our study highlights the need to identify the cytokine concentration range specific to the patient population with the disease of interest when used as a prognostic marker to predict outcomes. Importantly, we have shown the enhanced predictive value of cytokine measurements grouped in quartiles (e.g. IL-10 and/or TNF) when considered together with clinical variables in identifying patients with SAB at risk for persistence and mortality. Our subgroup analysis comparing MSSA vs MRSA bacteremia confirmed the enhanced predictive value observed above for the overall study population upon addition of either IL10 or TNF to the models, though the predictive value for the MRSA subgroup for persistence did not change with addition of Day 1 cytokine levels likely due to the limited sample size.

Notably, we observed a correlation with empiric vancomycin and beta-lactam combination therapy against development of persistent bacteremia. Others have shown that the combination of vancomycin and a beta-lactam agent to possess synergistic activity against *S. aureus* in vitro [17–19] and to reduce time to bacterial clearance in MRSA bacteremia [20, 21]. However, in a subset of patients, combination therapy has been associated with an increased risk of acute kidney injury and has no clear association with reduction in mortality [22]. Our findings could have practical applications in the management of patients with SAB. Measurement of either IL-10 or TNF at onset along with clinical risk factors may potentially help identify patients at risk for developing persistence and thus aid clinicians in deciding whether or not to initiate combination vancomycin and beta-lactam therapy empirically. Additionally, a measured level of either IL-10 or TNF in Quartile 4 identifies those at risk for death which provides a quantitative marker that together with clinical indicators could help prompt clinicians to re-evaluate management of SAB and source control measures as appropriate. Importantly, persistent growth of *S. aureus* in the blood may reflect a state of immunoparalysis that hinders bacterial clearance [23]. A quantitative biomarker measurement that is predictive of mortality can aid in the design of future biomarker-informed clinical trials to test the therapeutic benefit of adjunctive immune activating therapy that could potentially correct the underlying dysregulated host immune response.

This study has several limitations. First, we analyzed a limited cytokine panel. Other groups have identified additional cytokines and proteins, such as IL-1RN and CCL2, and found a significant association with persistent SAB and mortality. Our current study has expanded the panel of cytokines to include IFNγ and IL-1β. While it is possible that other biomarkers not included in this current study could also serve as prognostic markers, IL-10 and TNF have consistently been found to be significant predictors of outcome in patients with SAB. Second, despite over 600 patients had at least one cytokine measurement, only a subset of patients (*n* = 241) with persistent bacteremia had Day 1 cytokine measurements. Additionally, while the overall mortality is low in our study population, we have shown strong predictive value in this study for persistence and mortality when cytokine measurements are considered along with clinical variables. Future studies involving patients with different source of *S. aureus* bacteremia associated with high risk of death should be performed to confirm our findings. Finally, given that the majority of infections were caused by methicillin-susceptible *S. aureus* and that worse outcome is associated with bacteremia due to MRSA compared to MSSA, future studies exploring how methicillin resistance might affect cytokine response leading to different outcomes is of interest.

In conclusion, our observational study of a large cohort of over 600 patients with *S. aureus* bacteremia who had cytokine measurements enable us to identify the specific cytokines and define the respective concentration ranges measured at onset and Day 4 of bacteremia that are strongly predictive of bacterial persistence and 30-day mortality.

The re-defined “abnormal” range for IL-10 and TNF serum concentrations during *S. aureus* bacteremia differs greatly from that published by laboratory references which were derived from concentrations measured in healthy volunteers. Pending confirmation from future biomarker-informed studies, these quantitative immune biomarker measurements early during the course of infection could potentially help clinicians to better identify patients who would benefit from empiric combination antibiotic therapy or aggressive management that may include adjunctive immunomodulatory therapy.

## Supplementary Information

**Additional file 1: Table S1.** MSSA Bacteremia Multivariable Predictive Model for Persistence: Clinical Variables Alone and Added Predictive Value of Day 1 Cytokines. **Table S2.** MSSA Bacteremia Multivariable Predictive Model for 30-day Mortality: Clinical Variables Alone and Added Predictive Value of Day 4 Cytokines. **Table S3.** MRSA Bacteremia Multivariable Predictive Model for 30-day Mortality: Clinical Variables Alone and Added Predictive Value of Day 4 Cytokines.

## Data Availability

The datasets used and/or analyzed during the current study are available from the corresponding author on reasonable request.
