# Supplementary material for: Cytokine measurements add value to clinical variables in predicting outcomes for Staphylococcus aureus bacteremia
Source: BMC Infect Dis. 2021 Apr 5;21:317. doi: 10.1186/s12879-021-06010-0 (PMC8022397; doi:10.1186/s12879-021-06010-0)
Supplement: Supplementary file 1 — Additional file 1: Table S1. MSSA Bacteremia Multivariable Predictive Model for Persistence: Clinical Variables Alone and Added Predictive Value of Day 1 Cytokines. Table S2. MSSA Bacteremia Multivariable Predictive Model for 30-day Mortality: Clinical Variables Alone and Added Predictive Value of Day 4 Cytokines. Table S3. MRSA Bacteremia Multivariable Predictive Model for 30-day Mortality: Clinical Variables Alone and Added Predictive Value of Day 4 Cytokines. [file 12879_2021_6010_MOESM1_ESM.docx]

**Appendix**

**Table 1 MSSA Bacteremia** **Multivariable Predictive Model for Persistence: Clinical Variables Alone and Added Predictive Value of Day 1 Cytokines**

| **Model (Sample Size)** | **Odds Ratio**  **(95% CI)** | **p-value** | **AUC**  **(95% CI)** | **p-value for AUC difference from clinical model** |
| --- | --- | --- | --- | --- |
| **Clinical variable model (159)** |  |  |  |  |
| Septic shock at onset of SAB | 4.107  (1.42. 11.90) | <0.01 |  |  |
|  |  |  | 0.579 (0.506 - 0.651) |  |
| **Added Day 1 cytokine variables individually as quartiles ^1^:** | | | | |
| **TNF (159)** |  |  |  |  |
| Q1: <9.46 | Ref |  |  |  |
| Q2: 9.46 - 19.18 | 2.131  (0.49, 9.25) | 0.49 |  |  |
| Q3: 19.18 - 39.84 | 3.028  (0.74, 12.46) | 0.82 |  |  |
| Q4: >39.84 | 9.296  (2.41, 35.85) | 0.0002 |  |  |
|  |  |  | 0.722 (0.625 - 0.818) | 0.001 |
| **IL-10 baseline (150)** |  |  |  |  |
| Q1: <12.68 | Ref |  |  |  |
| Q2: 12.68 - 32.62 | 2.182  (0.50, 9.52) | 0.55 |  |  |
| Q3: 32.62 - 122.50 | 3.950  (0.98, 15.90) | 0.30 |  |  |
| Q4: >122.50 | 6.702  (1.66, 27.05) | 0.01 |  |  |
|  |  |  | 0.725 (0.628 - 0.821) | 0.003 |

**Table 2. MSSA Bacteremia** **Multivariable Predictive Model for 30-day Mortality: Clinical Variables Alone And Added Predictive Value of Day 4 Cytokines**

| **Model (Sample Size)** | **Odds Ratio**  **(95% CI)** | **p-value** | **AUC**  **(95% CI)** | **p-value for AUC difference from clinical model** |
| --- | --- | --- | --- | --- |
| **Clinical variable model (309)** |  |  |  |  |
| Septic shock at onset of SAB | 4.57  (1.35, 15.40) | 0.01 |  |  |
| PBS >=4 (vs. <4) | 5.19  (1.62, 16.65) | <0.01 |  |  |
| Tmax >38.3 at 72 hr after abx | 2.92  (0.86, 9.95) | 0.09 |  |  |
|  |  |  | 0.798 (0.696 - 0.901) |  |
| **TNF (309)** |  |  |  |  |
| Q1: <6.68 | Ref |  |  |  |
| Q2: 6.68 - 12.48 | 2.604  (0.25, 26.73) | 0.73 |  |  |
| Q3: 12.48 - 23.75 | 4.554  (0.50, 41.48) | 0.43 |  |  |
| Q4: >23.75 | 8.245  (0.99, 68.55) | 0.02 |  |  |
|  |  |  | 0.880  (0.825, 0.935) | 0.02 |
| **IL-10 (285)** |  |  |  |  |
| Q1: <5.93 | Ref |  |  |  |
| Q2: 5.93 - 13.61 | 0.529  (0.08, 3.70) | 0.19 |  |  |
| Q3: 13.61 - 28.21 | 0.860  (0.15, 5.00) | 0.54 |  |  |
| Q4: >28.21 | 4.629  (1.13, 18.96) | <0.001 |  |  |
|  |  |  | 0.878 (0.819 - 0.936) | 0.03 |

**Table 3**. **MRSA Bacteremia** **Multivariable Predictive Model for 30-day Mortality: Clinical Variables Alone And Added Predictive Value of Day 4 Cytokines**

| **Model (Sample Size)** | **Odds Ratio**  **(95% CI)** | **p-value** | **AUC**  **(95% CI)** | **p-value for AUC difference from clinical model** |
| --- | --- | --- | --- | --- |
| **Clinical variable model (197)** |  |  |  |  |
| Septic shock | 4.376  (1.43, 13.38) | <0.01 |  |  |
| PBS >=4 (vs. <4) | 9.522  (3.207, 28.727) | <0.0001 |  |  |
|  |  |  | 0.801  (0.690, 0.912) |  |
| **TNF (197)** |  |  |  |  |
| Q2: 6.82-13.06 | Ref |  |  |  |
| Q3: 13.06-26.31 | 1.119  (0.259, 4.828) | 0.88 |  |  |
| Q4: >26.31 | 1.094  (0.256, 4.681) | 0.90 |  |  |
|  |  |  | 0.840  (0.739, 0.941) | 0.07 |
| **IL-10 (183)** |  |  |  |  |
| Q2: 5.15-13.57 | Ref |  |  |  |
| Q3: 13.57-31.13 | 2.296  (0.339, 15.558) | 0.39 |  |  |
| Q4: >31.13 | 6.388  (1.111, 36.74) | 0.04 |  |  |
|  |  |  | 0.889  (0.811, 0.967) | 0.03 |
